# Evidence-Informed Development of a Bundle for Peripheral Intravenous Catheterization in Portugal: A Delphi Consensus Study

**DOI:** 10.3390/nursrep12030047

**Published:** 2022-07-07

**Authors:** Paulo Santos-Costa, Filipe Paiva-Santos, Liliana B. Sousa, Rafael A. Bernardes, Filipa Ventura, Anabela Salgueiro-Oliveira, Pedro Parreira, Margarida Vieira, João Graveto

**Affiliations:** 1 The Health Sciences Research Unit: Nursing, Nursing School of Coimbra, 3004-011 Coimbra, Portugal; filipesantos@esenfc.pt (F.P.-S.); baptliliana@esenfc.pt (L.B.S.); rafaelalvesbernardes@esenfc.pt (R.A.B.); filipaventura@esenfc.pt (F.V.); anabela@esenfc.pt (A.S.-O.); parreira@esenfc.pt (P.P.); jgraveto@esenfc.pt (J.G.); 2 Instituto Ciências da Saúde, Universidade Católica Portuguesa, 4169-005 Porto, Portugal; mmvieira@ucp.pt; 3 Centre for Interdisciplinary Research in Health, Universidade Católica Portuguesa, 4169-005 Porto, Portugal

**Keywords:** catheterization, peripheral, nurses, bundle, consensus

## Abstract

Contrary to many international settings, there are no clinical guidelines for peripheral intravenous catheter (PIVC) insertion and maintenance in Portugal. We sought to derive an international consensus on a PIVC bundle that could guide Portuguese nurses’ clinical decision-making in this scope. Methods: Two international vascular access specialist groups participated in an online Delphi panel. During the first round, specialists (*n* = 7) were sent a summary report from a previous observational study conducted in a surgical ward in Portugal. Based on the report findings, specialists were asked to provide five to eight PIVC insertion and maintenance interventions. Then, another set of specialists (*n* = 7) scored and revised the recommendations until a consensus was reached (≥70% agreement). The PIVC bundle was made available and discussed with the surgical ward’s nurses. Results: After three rounds, a consensus was achieved for five evidence-informed interventions: (i) involve the person and assess the peripheral venous network; (ii) maintain an aseptic no-touch technique; (iii) ensure proper catheter dressing and fixation; (iv) perform catheter flush & lock; (v) test the peripheral venous catheter’s functionality and performance at each shift. Conclusion: The final version of the PIVC bundle achieved consensus among international experts. Despite the positive feedback provided by the ward nurses, future studies are warranted to assess its effectiveness in standardizing PIVC care delivery and its potential implications for care outcomes in Portuguese clinical settings.

## 1. Introduction

Despite its ubiquity, peripheral intravenous catheterization (PIVC) remains one of the most challenging invasive procedures performed worldwide [1]. Recent data suggests that up to two-thirds of all PIVCs are removed prematurely due to complications such as phlebitis, infiltration, occlusion, or bloodstream infection [2,3,4]. 

This is a major concern for healthcare professionals since most hospitalized patients require long periods of intravenous administration, and PIVC failure can lead to substantial treatment delays [5]. Moreover, premature failure translates into new puncture attempts until successful insertion, damaging patients’ venous network, undermining their care experience, and increasing care costs [6,7]. This has led the Emergency Care Research Institute to list peripheral vascular harm as one of the major patient safety concerns in the world [8]. 

Several quality improvement measures in this field (e.g., nationwide standards of care, certified training courses on vascular access, and the existence of vascular access teams) have been reported in countries such as Spain, France, Germany, the United Kingdom, Italy, United States of America, and Australia [9,10,11,12,13,14,15]. However, despite having one of the top national healthcare systems globally, this is still not the case in Portugal. 

Previous authors have identified that the lack of an evidenced-informed standard of care could partially explain the unstandardized practices reported across the country [16,17,18,19]. This is especially true for Portuguese nurses, who are in charge of all aspects of PIVC care in the country, from device insertion to maintenance and surveillance. [17]. 

While developing a national guideline requires substantial resources and public governmental support, other evidence-informed options are available for local implementation [20]. A clinical bundle refers to a set of interventions supported by high-level evidence and defined for a specific clinical cohort and setting [21]. Clinical bundles are expected to be applied uniformly by healthcare professionals following an “all or none” approach. 

Previous studies have described the development and effectiveness of implementing PIVC bundles in specific wards, departments, or entire hospitals with positive results in decreasing complications such as phlebitis and catheter-related bloodstream infections [22,23,24]. After conducting a comprehensive systematic review [23], Ray-Barruel and colleagues reported that synthesizing evidence on PIVC bundle effectiveness was difficult due to high heterogeneity between studies relating to the reported bundle components, endpoints, time periods, definitions and reporting measures. This may be partially explained because bundle elements should be descriptive, defined for a specific patient population in one location, and allow for local customization and appropriate clinical judgment [21]. 

Thus, as part of a large action-research project in one of the largest oncology hospitals in Portugal, we sought to derive an international consensus on a bundle that could guide nurses’ clinical decision-making during PIVC insertion and maintenance. 

## 2. Materials and Methods

### 2.1. Study Design

We utilized a modified and anonymous internet-based Delphi approach to design the major components of a PIVC bundle between February and July 2021. 

The Delphi technique is a well-established, iterative, multi-stage procedure for reaching an agreement that employs at least two rounds of anonymous surveys [11]. The Delphi technique has been used before in vascular access studies with very specific intents, such as creating a vascular access minimum dataset, developing a global rating scale of ultrasound-guided vascular access competence, or identifying hemodialysis nurse-sensitive indicators [25,26,27]. 

The Delphi technique’s advantages include inviting many international participants who provide their opinions anonymously and with equal relevance. Furthermore, the study team chose this approach as a realistic and low-cost solution for bringing specialists together during the COVID-19 pandemic.

### 2.2. Study Procedures

**Figure 1:**
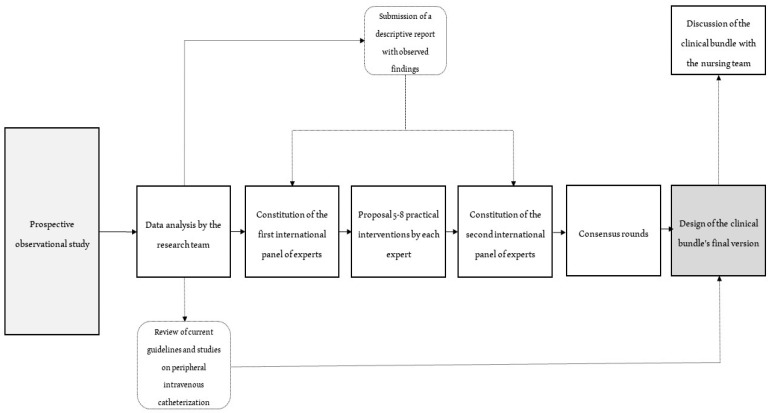
Overall study design.

First, the research team wrote a brief report summarizing findings from an observational study conducted in a surgical ward from the Instituto Português de Oncologia de Coimbra (Coimbra, Portugal) [28]. The selected nursing team participated in an action-research project that aimed to improve their peripheral intravenous catheterization practices and achieve better patient outcomes. The study procedures are represented in Figure 1.

Email invitations were sent to well-established international vascular access specialists (*n* = 25) to participate in the Delphi consensus study in two phases. In the first phase, specialists (*n* = 15) were invited to review the brief report sent by the research team and write five to eight evidence-informed interventions (EII) that could integrate a PIVC insertion and maintenance bundle that would later be implemented on the surgical ward. Specialists were informed of a few formal requirements before suggesting specific EII [21], namely: (i) the proposed interventions should be informed by recent evidence rather than from subjective experience, supported in international guidelines or studies of high methodological quality; (ii) each bundle intervention should be relatively independent; (iii) the PIVC bundle interventions should be descriptive and allow for local customization and appropriate clinical judgment. 

After this initial round, invitations were sent to a new group of specialists (*n* = 10). The second group was requested to review the same report and the proposed EIIs, rating and revising them through rounds until consensus is achieved. Two different approaches were used to collect feedback: (i) by answering “yes” or “no” to the question “Do you agree with the inclusion of this EII in the final version of the PIVC bundle?”; (ii) by rating their accordance with the writing of each EII, using a 7-points Likert scale ranging from 1 (completely disagree) to 7 (completely agree), where a score of four was considered a neutral stance. Specialists were able to explain their rationale and provide revisions to each EII.

At the end of each round, two research team members independently reviewed the specialists’ feedback to mitigate potential selection or judgment bias. The proposed changes to each EII (e.g., wording, merging similar interventions, exclusion) were discussed between the two researchers. Any discrepancies or disagreements were solved through the involvement of a third element of the research team. In succeeding rounds, participants were given a summary of the panel’s replies. Consensus was considered achieved when at least 70% of the specialists agreed to include an EII, and no major revisions were proposed (median Likert scale score ≥ 5 points).

After the PIVC bundle was completed, to avoid a more prescriptive nature and allow for appropriate clinical judgment [20], the research team included short guiding statements for each EII based on existing evidence in international guidelines and standards of care [29,30,31]. Likewise, as a matter of external validation and to ensure the PIVC bundle’s relevance and clinical applicability, the research team conducted an online session to present and discuss the results of the Delphi consensus study with the surgical ward’s nurses and manager. Given the nature of the ongoing action-research project, the clinical bundle would only be implemented at the surgical ward if the nursing team perceived it as a useful contribution for their daily clinical practice.

### 2.3. Participants

There is no standard panel size, nor has it been established what constitutes a large or small panel, although a minimum of ten experts is deemed appropriate [32]. The research team recruited well-known international specialists on vascular access using selective sampling. The lead researcher approached specialists via their institutional email and presented the ongoing study and its goals.

Specialists were selected based on the following criteria: (i) vast clinical or research experience in the field of vascular access, proved by their participation in working groups or vascular access associations; (ii) have published at least two scientific articles in the field of vascular access since 2019; (iii) show interest and availability to participate in the Delphi panel; (iv) be proficient in written English. 

### 2.4. Statistical Analysis

Successive surveys were administered online via Google Forms. Quantitative data analysis was performed using SPSS Statistics® (version 24, IBM SPSS; Chicago, IL, USA), using medians, interquartile ranges (IQR), frequencies, and percentages as descriptive statistics. 

### 2.5. Ethics

The primary study received a favourable review by the hospital’s Ethics Committee (ref. T.I. 24/2019, approved on 19 September 2019). Given the study design, written consent was waived for specialists as participation implied consent and all input was given anonymously, without interference by the research team. Study findings are reported according to the recommendations for the Conducting and Reporting Delphi Studies (CREDES) [33].

## 3. Results

Concerning round one, seven out of 15 international specialists on vascular access accepted participation in the Delphi panel (46.7% response rate). The main reasons for declining participation in the first round of the Delphi panel were “time unavailability” (*n* = 7) and “unfamiliarity with local challenges” (*n* = 1). Regarding the following rounds of the Delphi study, seven out of 10 invited international specialists also agreed to participate (70% response rate). Only one specialist claimed “time unavailability” as the main reason not to participate in the panel, while the remaining two specialists did not reply to the invitation. 

Detailed sociodemographic, academic, and professional data on the panels can be found in Table 1. Specialists were recruited from Portugal (*n* = 2), Spain (*n* = 3), The Netherlands (*n* = 2), the United Kingdom (*n* = 5), Australia (*n* = 1) and Brazil (*n* = 1). Most specialists (78.6%) reported being affiliated with a local or international association/network focused on vascular access care, research, and training.

### 3.1. Delphi Panel: Round One

**Figure 2:**
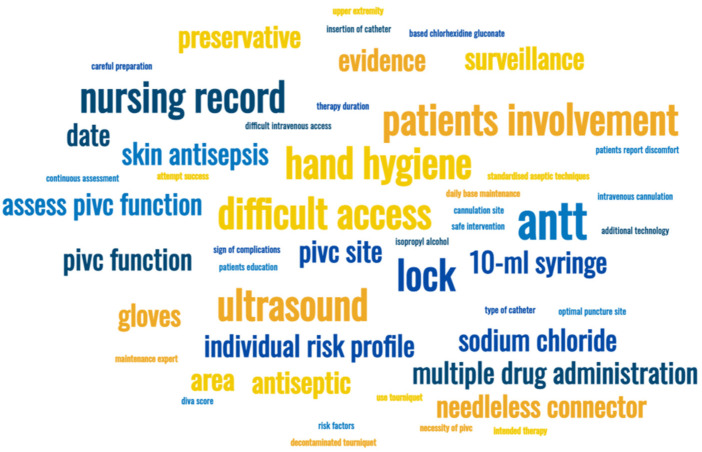
Word map reflecting the initial item pool.

After reviewing the report sent by the research team, the vascular access specialists proposed a total of 49 interventions that mirror their beliefs of essential care steps that Portuguese nurses should perform to overcome current quality and safety challenges. Although some shared a mutual focus, these proposed recommendations varied greatly in the terminology and sentence structure (Figure 2). 

The most recurrent themes found in the initial item pool concerned patient involvement throughout care delivery (6/49, 12.2%), the performance of skin antisepsis (5/49, 10.2%) and adherence to the aseptic no-touch technique (5/49, 10.2%), performance of catheter flushing before each use and between multiple drug administrations (4/49, 8.2%) and need to conduct an initial risk assessment before attempting PIVC insertion (4/49, 8.2%). On the other hand, the specialists only mentioned interventions such as glove use, team communication/collaboration or the need for specialists in PIVC insertion and maintenance once.

### 3.2. Delphi Panel: Rounds Two and Three

Before round two, given the similar focus of many of the recommendations, the research team organized them into 16 EIIs. The interventions were then sent to the new specialist panel for assessment. Only one EII was consensual during this round, which focused on the importance of early patient involvement and continuous education (EII1; 71.4% of agreement). Out of the 16 EII grouped by the research team, five were deemed important by up to two specialists (Table 2).

Still, EII15 (team communication & collaboration) and EII16 (insertion and maintenance experts) were not considered essential by any specialists. According to the provided feedback, specialists believed that both EIIs addressed important but out-of-scope interventions that would be hard to measure individual compliance. The specialists considered that communication and collaboration between the nursing team and creating opportunities for specialist nurses on vascular access are challenges that healthcare managers must address and may require the revision of institutional policies.

At the end of round two, the specialists provided valuable feedback on the listed EIIs. For example, EII1, EII2 and EII9 were considered to address the same challenge in Portuguese clinical settings. According to the experts, early patient involvement (EII1) also includes assessing the peripheral venous network and any previous experience of difficult intravenous access (DIVA). When nurses identify a moderate to high risk of DIVA (EII2), vein-locating technologies should be used to enhance the chances of first-attempt success (EII9). Patients should then be educated (EII1) about potential complications and strategies to avoid them during the fulfilment of their activities of daily living (e.g., showering, eating, dressing). These conclusions lead the research team to merge these elements into a single, broader EII (Table 2).

Merging some of the proposed EIIs positively affected specialists’ opinions since, at the end of the third round, the five EIIs were deemed consensual (with agreement rates ranging between 71.4% and 100%). At this stage, some levels of disagreement (M ≥ 6; IQR ≤ 2) were less focused on the proposed intervention but mostly on the wording revisions. Given that the panel consisted of specialists from different cultural and linguistic backgrounds, and translation from English to European Portuguese would solve most wording issues, we concluded the Delphi consensus-building process. Nevertheless, specialists were informed that if potential applicability and relevance issues were raised during its implementation in the surgical ward, the research team would carry out new consensus-building rounds. 

The agreed five EIIs emphasized the importance of: (i) involving the person and assessing the peripheral venous network; (ii) preserving the aseptic no-touch technique at all times; (iii) guaranteeing quality catheter dressing and securement; (iv) performing PIVC flush and lock; (v) assessing the PIVC’s integrity and functionality, at least once per shift. 

**Figure 3:**
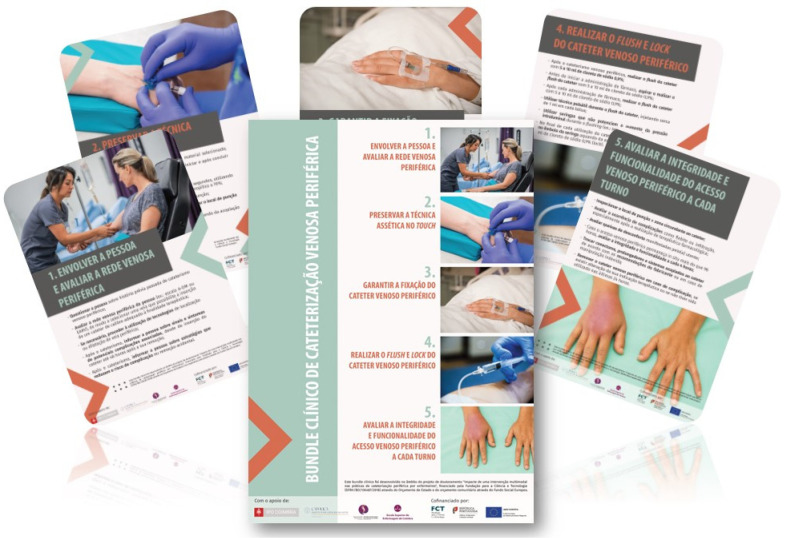
The graphic representation of the developed PIVC bundle.

To enhance the PIVC bundle’s applicability and ease of use, the research team included guiding recommendations for each EII. The bundle’s content was then translated from English into European Portuguese by two independent native speakers with a background in health sciences and vascular access to ensure the correct use of clinical terminology. During this process, no major challenges were faced concerning the bundle components’ semantic, conceptual, experiential, and idiomatic equivalence. With a graphic designer’s help, the research team developed the visual representation of the PIVC bundle (Figure 3).

### 3.3. Introducing and Reviewing the Bundle in a Clinical Setting

In July 2021, the research team held an hour-long advance discussion panel with thirteen surgical ward nursing team members. First, the previous observational study results were shared, and key areas of improvement were discussed. During this time, nurses emphasized that taking part in this action-research project was “an extremely interesting challenge that will lead to better clinical practices” (Specialist Nurse, Female, 14 years of experience) and “helps the nursing team to decrease the current rates (of complications) drastically” (Specialist Nurse, Female, 35 years of experience). 

The lead researcher then presented the PIVC bundle, discussing the proposed EIIs and guiding recommendations. Overall, nurses deemed the PIVC bundle an easily understandable and informative tool containing state-of-the-art evidence on PIVC insertion and maintenance care. All nurses found that the guiding recommendations addressed current challenges identified during the observational study. No difficulties were reported in interpreting the bundle. 

After the advance discussion panel, in collaboration with the ward manager, the research team made the PIVC bundle available to staff through visual posters and pocket-size laminated prints for individual use. 

## 4. Discussion

Evidence-informed nursing has gained momentum in recent years, challenging the paradigm of what should be the interlined relationship between practice, research, and education [34,35]. Promoting evidence-informed practice provides significant opportunities for nursing care to be more efficient and dynamic, maximizing clinical judgment [34,35]. Thus, efforts must be made to support and inform nurses’ decision-making according to the best scientific evidence available.

This challenge is particularly significant for Portuguese nurses when focusing on PIVC-related care. Over the last ten years, previous authors have addressed the scarcity of public-endorsed guidelines and standards of care in vascular access in the country [16,35,36]. Existing guidance is often developed locally by each ward/department’s nursing team, referencing outdated studies and without periodic revisions [36].

While developing public-endorsed guidelines can be a time and resource-consuming process, clinical bundles have emerged as a complementary approach that fosters evidence-based decision-making in clinical practice. Clinical bundles are significant for nursing practice since they should be designed to reflect challenges experienced in specific settings and with specific patient cohorts. 

After conducting a comprehensive systematic review on the effectiveness of PIVC bundles, Ray-Barruel and colleagues [23] found that the “majority of papers in this review reported consulting evidence-based guidelines before compiling and implementing a PIVC bundle”, while the remaining studies presumably based their interventions on local opinion or need. This defeats the purpose of a clinical bundle, which should be a short set of interventions that are already recommended in (inter)national guidelines, developed in consensus by a group of multidisciplinary clinicians.

In Portugal, a previous study conducted in an emergency room in the Alentejo region aimed to develop and implement a PIVC bundle to prevent catheter-related bloodstream infections [37]. However, the proposed bundle was derived from the author’s subjective judgement after consulting international guidelines and standards of care, omitting the importance of multidisciplinary and decentralized development to avoid bias. 

To the best of our knowledge, this study was the first conducted in Portugal that developed a data-supported and evidence-informed PIVC bundle, with the continuous involvement of specialists in vascular access. The resulting bundle proposes a set of five independent interventions that must be implemented to guarantee the delivery of safe and quality nursing care to patients who require peripheral venous access. The proposed EIIs tackle different challenges from PIVC insertion and maintenance, ranging from core nursing competencies (e.g., the importance of early patient involvement and education) to more practical interventions (e.g., preserving aseptic no-touch technique or catheter flushing and locking). Such measures appear to address current difficulties found in the surgical ward [28], which were expressed not just by ward nurses during focus groups but also through observation of current bedside practices. This might partially explain why the ward nurses had no reservations about its clinical usefulness or long-term implementation after the presentation session. 

Regardless, long-term adoption of the PIVC bundle will not be feasible after a one-time presentation, and ongoing strategies between the research team and the surgical ward’s nursing manager will be required. Previous studies have employed strategies similar to the ones used by the research team, such as lectures and discussion sessions (with a strong visual component), posters and individual booklets/leaflets [23]. However, these strategies are unidirectional, undermining action research’s true nature and purpose [38]. More engaging and innovative strategies have been reported, such as nominating patient care champions, slogan contests, alert stickers, and revising current nursing charts to reflect the bundle’s EIIs [23]. Regardless, the nursing team must be supported throughout the process and learn how to continuously execute the PIVC bundle through a systematized improvement plan [21,33]. 

Finally, it is only possible to determine long-term bundle adoption if recurrent compliance assessments are performed, requiring a clear understanding between the research and nursing teams of the pursued goals and what constitutes an improvement. Methodological recommendations on bundle development and implementation suggest an “all-or-none measurement, with a goal of 95 per cent or greater” [21], demanding that all bundle components are performed to be considered compliant (unless clinically contraindicated). 

Future discussions between the research and nursing teams must also focus on which effectiveness outcomes should be monitored. Numerous studies have focused on bloodstream infections as the main outcome [23], although infiltration and phlebitis were the main PIVC-related complications reported in the surgical ward [28]. However, aside from its potential effectiveness in reducing PIVC-related complications, we believe that continuous compliance may likely enhance nurse–patient communication and the first-attempt success rate. Thus, it becomes increasingly significant that future developments also focus on the PIVC bundle’s effect on patient-reported outcomes (PROMs) and patient-reported experience measures (PREMs) [39].

### Limitations

Our findings must be discussed within some limitations. First, this study reports the development of a PIVC bundle tailored for a specific setting and patient cohort, based on previously collected data at a surgical ward in Portugal. While developing tailored clinical bundles is the standard, this undermines the potential generalization of future findings to other wards or departments in Portugal. Although some of the proposed EIIs are universally regarded as essential, future authors must consider revising the proposed bundle to reflect challenges faced in a particular setting and patient cohort. Nonetheless, this study offers a structured approach to developing an evidence-informed PIVC bundle, constituting a foundation for other nursing teams in Portugal who wish to promote evidence-informed practice in this field.

Secondly, Delphi consensus studies are not immune to bias due to the specialists’ feedback or moderator effect. During this study, several steps were taken to reduce the chance of influencing the specialists’ judgement (e.g., more than one group of specialists was involved in the study; specialists’ feedback was reviewed independently by two members of the research team), and there were no conflicts of interest reported by any of the parties involved.

Lastly, the decision to conclude the consensus-building Delphi after three rounds may have influenced the quality of the PIVC bundle. Three decisions leaned in favor of this decision: (i) the two different measures used to define “consensus”; (ii) the study’s six-month duration, which could lead to participant tiredness and eventual dropout, affecting the quality and accuracy of their collected feedback; (iii) if the PIVC bundle was not well-accepted by the nursing team, specialists were informed that new rounds would be conducted. However, the number of conducted rounds seems to be on par with most Delphi studies reported in the literature, regardless of the field and goal [33].

## 5. Conclusions

Based on constant feedback from worldwide vascular access specialists and a comprehensive review of international standards of care, this study has developed consensus-based recommendations for a PIVC bundle. This study is the first in Portugal to employ a systematized methodological approach and use decentralized feedback to design a PIVC bundle. Despite the nurses’ positive review of the developed bundle, more research is needed to determine its impact on standardizing PIVC insertion and maintenance practices and reducing adverse events.

## Figures and Tables

**Table 1:** Characterization of the Delphi panel members.

Variables	Specialists	
	Round 1 (*n* = 7)	Rounds 2–3 (*n* = 7)
Age	M = 45 years (min. 33–max. 64)	M = 44 years (min. 37–max. 55)
Sex		
Male	3 (42.9%)	2 (28.6%)
Female	4 (57.1%)	5 (71.4%)
Scientific Background		
Medicine	1 (14.3%)	2 (28.6%)
Nursing	6 (85.7%)	5 (71.4%)
Highest academic title		
Master’s Degree	3 (42.9%)	3 (42.9%)
PhD	4 (57.1%)	4 (57.1%)
Professional experience	M = 21 years (min. 12–max. 45)	M = 23 years (min. 14–max. 39)
Current professional setting		
Clinical setting	3 (42.9%)	3 (42.9%)
Academia and/or research lab	3 (42.9%)	4 (57.1%)
Independent Consultant	1 (14.3%)	0 (0%)

Note. M = median; min. = minimum; max. = maximum.

**Table 2:** Characterization of the Delphi panel members.

Recommendations (Categorized)	Round 1	Round 2	Round 3
Patient involvement	6	EII 1 M = 5.0 (IQR = 1.0) RoA = 71.4%	EII 1 M = 6.0 (IQR = 1.0) RoA = 100%
Patient education	3		
Risk assessment	4	EII 2 M = 4.0 (IQR = 2.0) RoA = 57.1%	
Skin antisepsis	5	EII 3 M = 5.0 (IQR = 1.25) RoA = 57.1%	EII 2 M = 6.0 (IQR = 2.0) RoA = 85.7%
Antiseptic type and use	1	EII 4 M = 2.0 (IQR: 2.25) RoA: 28.6%	
Tourniquet type and use	1	EII 5 M = 2.0 (IQR = 1.25) RoA: 28.6%	
Aseptic no-touch technique	5	EII 6 M = 4.0 (IQR = 1.25) RoA = 57.1%	
Catheter flushing	4	EII 7 M = 4.0 (IQR = 2.25) RoA: 57.1%	EII 4 M = 6.0 (IQR = 1.0) RoA = 85.7%
Catheter lock	1		
Flushing solution and technique	2	EII 8 M = 3.5 (IQR = 1.0) RoA: 42.9%	
Use of vein-locating technology	1	EII 9 M = 2.0 (IQR = 1.25) RoA: 28.6%	Integrated into EII 1
Hand hygiene	2	EII 10 M = 2.0 (IQR = 1.25) RoA: 28.6%	Integrated into EII 2
Glove use	1	EII 11 M = 2.0 (IQR = 1.0) RoA: 14.3%	
Catheter dressing	3	EII 12 M = 4.0 (IQR = 1.25) RoA = 57.1%	EII 3 M = 6.0 (IQR = 1.0) RoA = 71.4%
Catheter securement	2		
Catheter maintenance (overall)	3	EII 13 M = 3.5 (IQR = 1.0) RoA = 57.1%	EII 5 M = 6.0 (IQR = 2.0) RoA = 85.7%
Catheter surveillance (complications)	3	EII 14 M = 4.0 (IQR = 1.25) RoA = 71.4%	
Team communication & collaboration	1	EII 15 M = 2.0 (IQR = 0.25) RoA = 0%	Excluded
Insertion and maintenance experts	1	EII 16 M = 2.0 (IQR = 0.25) RoA: 0%	Excluded
Total items	49	16	5

Note. EII = Evidence-informed interventions; M = median; IQR = Interquartile range; RoA = Rate of Agreement.

## Data Availability

The data presented in this study are available on request from the corresponding author.
